# Validation and further insight into the International Severe Asthma Registry (ISAR) eosinophil gradient algorithm in the Wessex AsThma CoHort of difficult asthma (WATCH) using historical blood eosinophil counts and induced sputum

**DOI:** 10.1111/cea.14109

**Published:** 2022-02-21

**Authors:** Clair Barber, Adnan Azim, Colin Newell, Aref Kyyaly, Hitasha Rupani, Hans Michael Haitchi, Peter Howarth, Ramesh Kurukulaaratchy

**Affiliations:** ^1^ School of Clinical and Experimental Sciences Faculty of Medicine University of Southampton Southampton UK; ^2^ National Institute for Health Research (NIHR) Southampton Biomedical Research Centre at University Hospital Southampton NHS Foundation Trust Southampton UK; ^3^ The David Hide Asthma & Allergy Research Centre St Mary’s Hospital Newport UK; ^4^ Biomedical Science Faculty of Sport, Health and Social Sciences Solent University Southampton Southampton UK; ^5^ Respiratory Department University Hospital Southampton NHS Foundation Trust Southampton UK; ^6^ Institute for Life Sciences University of Southampton Southampton UK

**Keywords:** asthma, clinical immunology, eosinophils, innate immunity, pneumology

**Key messages**

- The eosinophil gradient algorithm from ISAR is successfully replicated in the WATCH cohort of difficult asthma.
- Repeating eosinophil measures in difficult asthma increases the probability of patients being considered likely to have an eosinophilic background.
- Contemporaneous blood and sputum measurements are proven theragnostic biomarkers. However, they underestimate the presence of underlying eosinophilic disease.

To the Editor,

Severe asthma is a heterogeneous disease comprising numerous endophenotypes.^1^ In recent years, there has been increased focus on eosinophils and type 2 (T2) inflammatory pathways as treatable traits in severe asthma, as these can be effectively targeted by an emerging portfolio of biologic therapies.^2^ Traditionally, the presence of increased eosinophils in induced sputum was used to define eosinophilic asthma. Sputum induction and analysis, however, require specialist expertise, preventing its use in routine clinical care. Consequently, the classification of severe eosinophilic asthma by blood eosinophil status has become commonplace in clinical practice.

There are, however, limitations in defining patients as eosinophilic or not, as severe asthma patients have high oral or inhaled corticosteroid burden which may suppress blood eosinophils and mask the underlying eosinophilic nature of the disease. Particularly if this trait is defined by limited timespan assessment in this variable disease.^1^, ^3^, ^4^ Consistent with this, we recently reported that repeated blood eosinophil counts (BECs) over time can help unmask the underlying T2 asthma status not revealed by a single snapshot measure.^3^ This longitudinal approach demonstrated a higher burden of T2 asthma status than previously reported. Thus, rigorous approaches are needed to correctly define non‐eosinophilic and eosinophilic forms of severe asthma to accurately guide clinical management.

Following our longitudinal analysis of blood eosinophilia in severe asthma, the International Severe Asthma Registry (ISAR) consortium recently presented a novel multidimensional algorithm to determine the probability of an eosinophilic phenotype within a severe asthma population.^5^ This algorithm combined BECs with relevant clinical indices (on anti‐ interleukin‐5 [IL‐5] or anti‐IL‐5Rα therapy, on maintenance oral corticosteroids [OCS], history of nasal polyps [NP], fraction of exhaled nitric oxide [FeNO] and adult‐onset asthma). The resulting probability of eosinophilic disease ranged from Grade 0 (unlikely) to Grade 3 (most likely). There were similarities between findings reported for this ISAR algorithm and our recent assessment of eosinophilic T2 difficult‐to‐treat/severe asthma status using longitudinal measures of BECs in the Wessex AsThma CoHort of difficult asthma (WATCH) study.^3^

The ISAR eosinophil gradient algorithm report identified two points that merit further understanding and which could be addressed by our WATCH cohort data: firstly, how longitudinal BEC assessment might consolidate these algorithm‐derived phenotypes; secondly, how the identification of eosinophilic asthma via the ISAR algorithm aligns with the traditional method of sputum analysis.

## 1 METHODS

Wessex AsThma CoHort of difficult asthma is a real‐world study of patients with difficult‐to‐treat/severe asthma attending a tertiary centre (Southampton, United Kingdom). The study had national research ethics committee (REC) approval (reference 14/WM/1226), and all patients provided written informed consent. The WATCH study protocol and methodology has been described previously.^6^ The WATCH dataset includes current and historic BEC data plus induced sputum data in a subset of patients with severe asthma. Sputum was induced in poorly controlled patients who were at least 4 weeks clear of an exacerbation. We applied the ISAR algorithm to the WATCH population to explore its generalizability, using both a single BEC taken at enrolment and, where available, observations of BECs up to 15 years prior. Additionally, we correlated induced sputum granulocyte phenotypes with the ISAR‐defined phenotype in a subset of WATCH patients.

## 2 RESULTS

Replication of the ISAR algorithm in 471 patients from the WATCH cohort confirmed the predominance of an eosinophilic status regardless of the BEC observation timeframe applied (Table 1). The weakest alignment in frequency of Grade 3 status between WATCH and ISAR was found when a single snapshot BEC measure was used: though 70% of patients considered at least “likely eosinophilic” (Grade 2), only 45% of the population being considered “most likely eosinophilic” (Grade 3). Progressively greater alignment in the proportions of ISAR Grades was found by increasing the retrospective reach of BEC observation in the WATCH cohort (Table 1), which increased the proportion of patients classified as Grade 3 and decreased the proportion of patients classified as Grade 0.

**TABLE 1:** Characterization of eosinophilic and non‐eosinophilic phenotypes of difficult asthma in the WATCH cohort using ISAR eosinophil gradient algorithm

Highest BEC Cells/µl	Treatment or clinical characteristics	Eosinophilic phenotype	ISAR study	WATCH (latest result test only)		WATCH (highest result in last 5 years)		WATCH (highest result in last 10 years)		WATCH (highest result ever)	
			(%)	*N* (%)	*N* (%)	*N* (%)	*N* (%)	*N* (%)	*N* (%)	*N* (%)	*N* (%)
≥300	n/a	Grade 3 Most likely	83.8%	163 (32.6%)	225 (45.0%)	296 (59.2%)	335 (67.0%)	342 (68.4%)	374 (74.8%)	359 (71.8%)	387 (77.4%)
n/a	On anti‐IL−5 or anti‐IL−5Rα therapy	Grade 3 Most likely		39 (7.8%)		15 (3.0%)		10 (2.0%)		8 (1.6%)	
150–300	Long‐term OCS	Grade 3 Most likely		15 (3.0%)		19 (3.8%)		18 (3.6%)		17 (3.4%)	
	Presence of >2 of the following NP, FeNO >25 ppb or adult onset (no long‐term OCS)	Grade 3 Most likely		8 (1.6%)		5 (1.0%)		4 (0.8%)		3 (0.6%)	
	Either NP, elevated FeNO or adult onset (no long‐term OCS)	Grade 2 Likely	3.9%	28 (5.6%)		29 (5.8%)		29 (5.8%)		25 (5.0%)	
	No NP, elevated FeNO, adult onset or long‐term OCS	Grade 1 Least likely	1.6%	28 (5.6%)		27 (5.4%)		29 (5.8%)		25 (5.0%)	
<150	Long‐term OCS	Grade 2	4.4%	71 (14.2%)		33 (6.6%)		22 (4.4%)		20 (4.0%)	
	Either NP, FeNO >25 ppb or adult onset (no long‐term OCS)	Grade 1 Least likely	4.7%	82 (16.4%)		39 (7.8%)		25 (5.0%)		22 (4.4%)	
	No NP, elevated FeNO, adult‐onset or long‐term OCS	Grade 0 Unlikely (non)	1.6%	66 (13.2%)		37 (7.4%)		21 (4.2%)		21 (4.2%)	

Note

Table replicated from the ISAR phenotypes Heaney 2021^5^ compared to the WATCH data.

FeNO, NP and mOCS were current at the time of enrolment.

Abbreviations: BEC, Blood eosinophil counts; FeNO, fraction of exhaled nitric oxide; IL‐5, interleukin 5’; ISAR, International Severe Asthma Registry; n/a, not applicable; NP, nasal polyps; OCS, oral corticosteroids; ppb, parts per billion; WATCH, Wessex AsThma CoHort of difficult asthma.

As in ISAR, no differences were observed between WATCH patients classified as Grade 2 or higher compared to patients classified as Grade 0 with regard to BMI (29.8 vs. 29.7 *p* = .625), presence of rhinitis (67.1% vs. 63.2% *p* = .915), asthma control (ACQ6 score 2.5 vs. 2.1 *p* = .354), or number of annual exacerbations (3.0 vs. 2.5 *p* = .452). In addition, as seen in ISAR, Grade 2 or higher patients were older (age at enrolment 55.0 vs. 40.0 *p* = .002), had a higher FeNO (20.3 vs. 11.0 *p* = .004) and had a later disease onset (age at asthma diagnosis 21.5 vs. 6.5 *p*=<.0001). Grade 0 patients were less likely than Grade 2 or higher to be male (9.5% vs. 37.3%, *p* = .019) and had better preserved lung function (median post‐BD FEV_1_% predicted 89.9% vs. 74.6% *p* = .05, median post‐BD FEV_1_:FVC ratio 80.0 vs. 67.0 *p* = .02). Unlike in ISAR, no difference was found in the prevalence of eczema ever (25.5% vs. 30.0% *p* = .853), atopy (67.6% vs. 68.4% *p* = .862) or median total IgE (89.7 vs. 50.9 *p* = .1).

Similar ISAR eosinophil phenotype distributions were observed in the subset (*n* = 130) of WATCH patients with paired sputum analysis: 107 (82.3%) patients were classified as Grade 3 (Table 2). Only 1 patient (0.8%) was classed as Grade 0; this patient presented with a pauci granular sputum phenotype (Table 2). When sputum eosinophilia (≥2% eosinophils) alone is used to define eosinophilic asthma, less than half of patients are found to be eosinophilic (36% patients with ≥2% sputum eosinophils and 12% with mixed granular sputum [≥2% eosinophils, ≥61% sputum neutrophils]). The pauci granular population had fewer patients reported to have CT scan evidence of bronchiectasis than those with sputum granular disease; however, no significant difference in the presence of bronchiectasis was identified between sputum neutrophilic and sputum eosinophilic patients. 46% of patients classified as Grade 3 did not have evidence of sputum eosinophilia.

**TABLE 2:** Sputum granulocyte distribution by eosinophilic phenotype

	Number (%)	Sputum snapshot presenting as			
		Eosinophilic (Eos ≥2%, Neut <61%)	Mixed granular (Eos ≥2%, Neut ≥61%)	Neutrophilic (Eos <2%, Neut ≥61%)	Pauci‐granular (Eos <2%, Neut <61%)
Sub cohort	130	47 (36%)	16 (12%)	17 (13%)	45 (35%)
Grade 3, most likely	107 (82.3%)	45 (42.1%)	13 (12.1%)	14 (13.1%)	35 (32.7%)
Grade 2 Likely	13 (10.0%)	2 (15.4%)	3 (23.1%)	5 (38.5%)	3 (23.1%)
Grade 1 Least likely	9 (6.9%)	0 (0.0%)	0 (0.0%)	3 (33.3%)	6 (66.7%)
Grade 0 Unlikely (non)	1 (0.8%)	0 (0.0%)	0 (0.0%)	0 (0.0%)	1 (100.0%)

Note

ISAR grade based on highest blood eosinophil count ever.

Abbreviations: Eos, Eosinophil; Neut, neutrophil.

## 3 DISCUSSION

We validated the ISAR eosinophil gradient algorithm in the WATCH cohort and demonstrated that extended longitudinal BEC monitoring within that framework increases the probability of identifying Grade 3 patients. It is notable that in both ISAR and WATCH datasets, a very high percentage of subjects were Grade 3 “most likely eosinophilic” using this algorithm. We previously showed remarkably similar overwhelming prevalence of underlying eosinophilic status in WATCH using an alternative perspective of longitudinal BEC monitoring. While there is potential selection bias with that latter approach (given increased propensity to perform blood counts when patients are exacerbating), the ISAR algorithm corroborates that observation by including clinical characteristics, which is particularly relevant when longitudinal BEC data are limited. When present, however, the inclusion of multiple historic BECs, mitigates against granulocyte count instability and treatment effects, in the detection of an underlying eosinophilic phenotype. Our WATCH data thus support Heaney et al's findings and those of others,^7^ that severe asthma is mostly an eosinophilic disease.^5^

It is worth noting that while the terms eosinophilic and T2 asthma are often applied interchangeably, the ISAR algorithm does not incorporate measures of atopic predisposition such as total IgE or specific allergen sensitization. Since allergy is a T2‐associated process that omission might raise concerns that the ISAR algorithm could miss a proportion of T2 patients. However, the ISAR algorithm still finds overwhelming prevalence of eosinophilic status. As no differences were identified in eczema, atopy or total IgE between patients considered Grade 2 and above vs. those considered Grade 0 these definitions cannot be considered interchangeable using the ISAR algorithm in the WATCH cohort.

Our sputum findings emphasize the value of longitudinal repeat measures in truly understanding the underlying phenotypic propensity and are consistent with a recent publication that also highlighted that single sputum measures underestimate the likelihood of being classified as eosinophilic.^4^ Variable granulocyte measures are also associated with poorer disease outcome,^3^, ^8^ highlighting the prognostic and diagnostic advantages of longitudinal repeat measurements. In addition, we are yet to fully understand the longitudinal stability of granulocyte phenotypes, the frequency of phenotype switching and how such events influence factors like disease severity and remission. We are also blinded to the influence granulocytes have on each other within this notoriously variable disease.

Importantly, the presence of an eosinophilic phenotype does not exclude the concomitant existence of additional biology, as 25% of those identified as eosinophilic by sputum measures in WATCH also had neutrophilic airways disease. Such additional biology is not apparent with the use of BEC for phenotypic classification. There thus remains an unmet need for peripheral blood biomarkers that reflect airway biology additional to that linked to eosinophilic inflammation. One such measure is Chitinase 3 Like 1 (CHI3L1/YKL‐40). Liu and colleagues identified higher measures of the T1 biomarker CHI3L1/YKL‐40 in serum of patients with normal sputum eosinophil levels,^9^ and CHI3L1/YKL‐40 was also identified in our previous severe asthma cohort as a sub‐phenotype in severe asthma.^1^ Thus, monitoring a broad spectrum of airway inflammatory markers in asthma should be an important future consideration in addition to the use of classifications like the ISAR algorithm.

In conclusion, though the term “non‐eosinophilic asthma” is used to describe severe asthma patients without current evidence of raised eosinophils while on high dose steroids. It is imprecise: many of these patients will have an underlying eosinophilic phenotype. Nevertheless, though, they may underestimate the presence of an eosinophilic phenotype. It is important to reiterate that contemporaneous blood and sputum measurements are proven theragnostic biomarkers, predicting response to anti IL‐5 and steroid treatment for reducing asthma exacerbations.

## CONFLICT OF INTEREST

Dr. Hitasha Rupani reports Speaker and consultancy fees from AstraZeneca, GlaxoSmithKline, Teva, Novartis and Chiesi. Professor Peter Howarth reports employment by GSK outside of the submitted work. Clair Barber, Adnan Azim, Colin Newell, Aref Kyyaly, Hans Michael Haitchi and Ramesh Kurukulaaratchy declare that they have no known competing financial interests or personal relationships that could have appeared to influence the work reported in this paper.

## AUTHOR CONTRIBUTION

CB, AA, CN, AK, HR, HMH, PH, RJK all contributed to study development, conduct and governance. AA performed the statistical analysis. CB wrote the first draft of the manuscript AA, CN, AK, HR, HMH, PH, RJK all contributed to manuscript development. RJK acts as guarantor for the manuscript. All authors have read and approved the manuscript before submission.
